# Recombinational Landscape and Population Genomics of *Caenorhabditis elegans*

**DOI:** 10.1371/journal.pgen.1000419

**Published:** 2009-03-13

**Authors:** Matthew V. Rockman, Leonid Kruglyak

**Affiliations:** 1 Lewis-Sigler Institute for Integrative Genomics, Princeton University, Princeton, New Jersey, United States of America; 2 Department of Ecology and Evolutionary Biology, Princeton University, Princeton, New Jersey, United States of America; 3 Department of Biology, New York University, New York, New York, United States of America; 4 Center for Genomics and Systems Biology, New York University, New York, New York, United States of America; 5 Howard Hughes Medical Institute, Chevy Chase, Maryland, United States of America; University of Chicago, United States of America

## Abstract

Recombination rate and linkage disequilibrium, the latter a function of population genomic processes, are the critical parameters for mapping by linkage and association, and their patterns in *Caenorhabditis elegans* are poorly understood. We performed high-density SNP genotyping on a large panel of recombinant inbred advanced intercross lines (RIAILs) of *C. elegans* to characterize the landscape of recombination and, on a panel of wild strains, to characterize population genomic patterns. We confirmed that *C. elegans* autosomes exhibit discrete domains of nearly constant recombination rate, and we show, for the first time, that the pattern holds for the X chromosome as well. The terminal domains of each chromosome, spanning about 7% of the genome, exhibit effectively no recombination. The RIAILs exhibit a 5.3-fold expansion of the genetic map. With median marker spacing of 61 kb, they are a powerful resource for mapping quantitative trait loci in *C. elegans*. Among 125 wild isolates, we identified only 41 distinct haplotypes. The patterns of genotypic similarity suggest that some presumed wild strains are laboratory contaminants. The Hawaiian strain, CB4856, exhibits genetic isolation from the remainder of the global population, whose members exhibit ample evidence of intercrossing and recombining. The population effective recombination rate, estimated from the pattern of linkage disequilibrium, is correlated with the estimated meiotic recombination rate, but its magnitude implies that the effective rate of outcrossing is extremely low, corroborating reports of selection against recombinant genotypes. Despite the low population, effective recombination rate and extensive linkage disequilibrium among chromosomes, which are techniques that account for background levels of genomic similarity, permit association mapping in wild *C. elegans* strains.

## Author Summary

*C. elegans* is a model system for diverse fields of biology, but its ability to serve as a model for quantitative trait gene mapping depends on its recombination rate in the laboratory and in nature. The latter is a function of how worms mate and migrate in the wild. We examined the patterns of recombination in a population that we put through thousands of meioses in the laboratory and in a collection of strains isolated from nature. The data suggest that meiotic recombination rate is highly regular in worms, with discrete domains whose boundaries we identify. The pattern in natural strains suggests that population structure, population size, outcrossing rate, and selection combine to suppress the overall effects of recombination. Moreover, some “wild” strains appear to be laboratory contaminants. Nevertheless, the history of recombination in wild worms is sufficient to permit correlations between genotype and phenotype to pinpoint the loci responsible for phenotypic variation.

## Introduction

The allelic variants that underlie heritable phenotypic variation are distributed along chromosomes. Their distribution is shaped by the machinery of meiosis within individuals and by mutation, selection, and drift among them. To discover the genetic basis of complex traits, and to understand the evolutionary dynamics that shape this genetic architecture, we must characterize empirical patterns of linkage and linkage disequilibrium. We have undertaken this task in the nematode *C. elegans*.

Mapping of thousands of mutants to the genome and molecular studies of meiotic machinery have provided a view of the large-scale landscape of the *C. elegans* recombination map. The chromosomes exhibit nearly complete crossover interference [1], such that each chromosome experiences one crossover per meiosis and has a genetic length of 50 cM [2]. Accumulated data from thousands of two- and three-point mapping crosses and small-scale SNP-based analyses have demonstrated a general pattern of large, nearly constant-rate domains on the autosomes, with high recombination in chromosome arms and low recombination in chromosome centers. Despite strong global regulation of crossover number, many details remain unclear, including the locations of the domain boundaries, the occurrence of fine-scale variation within domains, and the existence of domain structure on the X chromosome. Moreover, evidence for the genetic control of crossover number and position [1]–[4] leaves open the possibility that segregating variants may influence recombination patterns in experimental crosses of natural isolates. Because recombination patterns have been studied only on broad scales in individual crosses, involving fewer than two dozen markers per chromosome, dense characterization of a massive cross promises to clarify the recombinational landscape.

*C. elegans* is one of the most exhaustively studied of all species with respect to developmental, behavioral, and physiological genomics, but studies of its population biology have lagged. Although natural genetic variation has been a source of alleles for genetic analysis in *C. elegans* since long before the system became a model [5], the widely accepted notion that worms exhibit little variation has discouraged investigations of their diversity. The difficulty of collecting *C. elegans* from the wild has compounded the problem. Nevertheless, recent work has revealed abundant heritable phenotypic variation among wild *C. elegans* strains [6]–[20] and has begun to reveal the ecological context for this species [16], [17], [21]–[25]. *C. elegans* geneticists have exploited this variation to map quantitative trait loci [26]–[37], and in a handful of cases to identify the causal mutations underlying phenotypic variation (in genes *npr-1*, *mab-23*, *tra-3*, *zeel-1*, *plg-1*, and *scd-2* [10], [30], [38]–[43]).

In parallel, studies of variation at molecular markers have begun to provide an account of the distribution of genetic variation within and among localities and across genomic regions [6], [7], [23], [24], [40], [41], [43]–[60]. These studies have shown that the species exhibits substantially lower levels of polymorphism and higher levels of linkage disequilibrium than other model systems, even those, like *Arabidopsis thaliana*, that share with *C. elegans* a primarily selfing mating system. The empirical pattern of linkage disequilibrium may result as much from selection against recombinant genotypes as from attributes of population biology such as population size and outcrossing rate [24],[61]. A genome-wide assessment of linkage disequilibrium is required to determine whether natural isolates of *C. elegans* will be useful for mapping loci by association.

We generated and genetically characterized a recombinant inbred advanced intercross population to gain insights into the recombination map in *C. elegans*, and we characterized a large panel of wild strains to characterize linkage disequilibrium. The data on recombination in the lab and in the wild reveal the role of population genomic processes in shaping genotypic diversity in *C. elegans*, and they lay the groundwork for rapid discovery of the genes underlying phenotypic variation.

## Results

### Patterns of Recombination in Recombinant Inbred Advanced Intercross Lines

We genotyped 1454 nuclear SNP markers in 236 recombinant inbred advanced intercross lines (RIAILs). These lines represent the terminal generation of a 20-generation pedigree founded by reciprocal crosses between the laboratory wild type strain N2 (Bristol) and the Hawaiian isolate CB4856. The pedigree includes ten generations of intercrossing (random pair mating with equal contributions of each pair to succeeding generations [62]) followed by 10 generations of selfing.

The SNP markers span 98.6% of the physical length of the chromosomes (Table S1). The median spacing is 61,160 bp, and 80% of intervals are shorter than 100 kb. Only 35 marker intervals (2.4%) are greater than 200 kb. The RIAILs contain 3,629 breakpoints in 772 marker intervals; some breakpoints may be identical by descent because of the shared ancestry during the intercrossing phase of RIAIL construction. An estimate of the mapping resolution of the panel, based on the distances between intervals containing breakpoints, yields a median bin size of 98 kb. Because larger bins contain more of the genome than smaller bins, the expected size of a bin in which a uniformly distributed QTL will fall is 225 kb.

The RIAILs exhibit a genetic map length of 1588 cM, a 5.3-fold expansion of the 300 cM F2 genetic map. The realized expansion is 93% of the expected 5.7-fold map expansion, a difference attributable, at least in part, to the action of selection during the construction of the lines.

**Figure 1:**
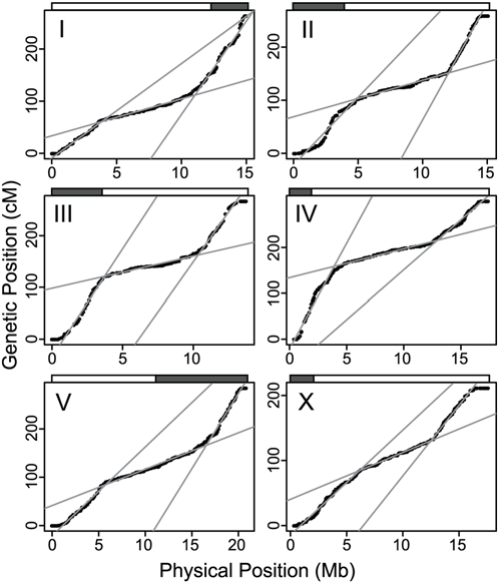
Recombination rate domains. Marey maps for each chromosome show genetic position of each marker (black points) as a function of physical position. Genetic position is measured in centiMorgans as defined on the recombinant inbred advanced intercross line population; these are not meiotic distances. Gray lines show the fits of segmented linear regressions, which estimate the boundaries of the recombination domains and their relative recombination rates. The shaded boxes above each plot show the genetically defined positions of the pairing centers [69].

Although selection and drift may alter the relationship between recombination fraction and meiotic recombination rate [63],[64], the observed recombination fractions are qualitatively informative about global patterns of recombination rate variation across *C. elegans* chromosomes. The genetic maps for the six *C. elegans* chromosomes are similar to one another and exhibit five distinct domains: two tips with effectively zero recombination, two high recombination arms, and a low recombination center, consistent with the pattern observed in classical two- and three-point mapping crosses [65]. These domains are evident in Marey maps [66], which show genetic position as a function of physical position (Figure 1; Table 1). As the recombination rate within each domain is relatively constant, we used a segmented linear regression to identify the boundaries between the domains.

**Table 1:** Chromosomal Domains.

Chr		left tip	left arm	center	right arm	right tip
I	Size (kb)	527	3331	7182	3835	197
	Size (%)	3.5	22.1	47.7	25.4	1.3
	Right end (kb)	527	3,858	11,040	14,875	15,072
	Rate^a^ (cM/Mb)	0	3.43	1.34	6.78	0
II	Size (kb)	306	4573	7141	2589	670
	Size (%)	2.0	29.9	46.7	16.9	4.4
	Right end (kb)	306	4,879	12,020	14,609	15,279
	Rate^a^ (cM/Mb)	0	4.92	1.33	8.47	0
III	Size (kb)	494	3228	6618	2877	567
	Size (%)	3.6	23.4	48.0	20.9	4.1
	Right end (kb)	494	3,722	10,340	13,217	13,784
	Rate^a^ (cM/Mb)	0	7.83	1.17	7.24	0
IV	Size (kb)	720	3176	9074	3742	782
	Size (%)	4.1	18.2	51.9	21.4	4.5
	Right end (kb)	720	3,896	12,970	16,712	17,494
	Rate^a^ (cM/Mb)	0	7.65	1.05	3.64	0
V	Size (kb)	643	5254	10653	3787	583
	Size (%)	3.1	25.1	50.9	18.1	2.8
	Right end (kb)	643	5,897	16,550	20,337	20,920
	Rate^a^ (cM/Mb)	0	3.22	1.32	5.47	0
X	Size (kb)	572	5565	6343	3937	1302
	Size (%)	3.2	31.4	35.8	22.2	7.3
	Right end (kb)	572	6,137	12,480	16,417	17,719
	Rate^a^ (cM/Mb)	0	3.81	1.70	5.14	0
ALL	Size (kb)	3262	25127	47011	20767	4101

a Rates are derived from the slopes of the segmented linear fits, scaled to yield a total genetic length of 50 cM for each chromosome.

The central domain of each autosome occupies roughly half the chromosome's length, despite the very different lengths of the chromosomes (Table 1). For example, the center of chromosome V is 10.7 Mb, 51% of the chromosome length, while the center of chromosome III is 6.6 Mb, 48% of that chromosome's length. Because all the centers have very similar rates of recombination per base pair (Table 1), their different physical lengths mean that the amount of recombination in each center (its genetic length) varies with total chromosome length. The constraint of one breakpoint per chromosome then requires that the amount of recombination in the arms of each chromosome varies inversely with chromosome length; shorter chromosomes have a larger fraction of their recombination events in their arms, and the physical sizes of the arms explain much of the variation among arms in recombination rates (*r^2^* = 0.51, p = 0.009). Nevertheless, the arms are heterogeneous in relative and absolute length and recombination rate, and the central domains are not perfectly centered on the chromosomes, consistent with the finding of Barnes et al. [65]. Most notably, the left arm of chromosome IV has a relative recombination rate more than twice that of the right arm, though they differ in size by only 15% (Figure 1; Table 1).

**Figure 2:**
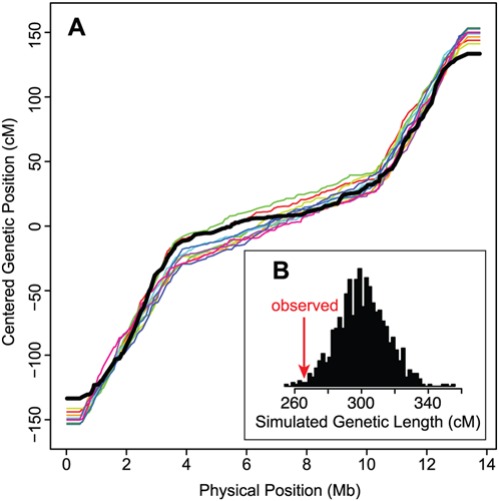
Simulated chromosomes. (A) The Marey maps for actual chromosome III data (black) and 10 chromosome III datasets simulated with discrete, constant-rate recombination domains (colors) show that variation within domains and indistinct boundaries between domains are expected. (B) The observed genetic length of chromosome III is smaller than expected. The histogram shows the lengths of 1000 chromosome III datasets simulated assuming one crossover per meiosis.

Inspection of the Marey maps suggests that there may be additional rate variation within the defined domains. To determine whether such variation is expected in the case of constant-rate domains, we simulated chromosomes along the RIAIL pedigree with discrete, constant-rate recombination domains, and we recorded the simulated genotypes at the same marker intervals as our actual genotype data. The simulated chromosomes exhibit patterns of variation within the discrete rate domains qualitatively similar to the observed data, preventing us from placing confidence in the fine-scale patterns in the data (Figure 2A). Nevertheless, the fine-scale variation observed in our data is largely concordant with that present in genetic maps derived from independent two- and three-point mapping crosses with classic visible markers (Figure S1), compiled in WormBase [67]. The general concordance between our map, derived from meioses at 25°C, and the WormBase map, which comes from crosses performed at various temperatures but primarily at 20°C, does not support the notion that the distribution of crossovers is strongly temperature dependent [68].

In our data, each chromosome has one very sharp center-arm boundary and one that is less sharp, and boundaries exhibit the identical pattern in the classical maps. In five of the six chromosomes, the less-sharp boundary is on the side of the chromosome that holds the pairing center [69] (Figure 1). The exception is chromosome III.

We find two points of disagreement between our results and previous discussion of recombination maps in *C. elegans*. First, the X chromosome clearly possesses domain structure similar to that of the autosomes (Figure 1), contrary to inferences from sparser data. The major distinguishing feature of the X-chromosome center is its relative size, 36% of the chromosome length, which is substantially less than the 47–52% on the autosomes. Second, we find that the chromosome tips have extremely low recombination rates; the terminal domain of each chromosome end is a region of effectively zero recombination, a pattern observed previously only for the right tip of the X [65] and more recently for chromosome III [68]. Every chromosome terminus contained a series of nonrecombining markers, and these domains ranged in size from 200 kb (IR) to 1300 kb (XR), averaging 600 kb.

### Selection

**Figure 3:**
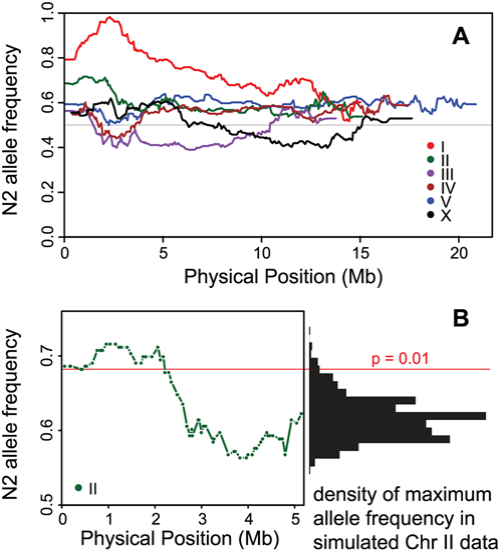
Allele frequencies in the recombinant inbred advanced intercross lines. (A) The frequency of the N2 allele at each marker along the chromosomes. The expected frequency, 0.5, is represented by the gray line. (B) A close view of the allele frequencies on the left side of chromosome II shows a significant skew toward N2 alleles. The histogram on the right represents the maximum allele frequency skew for 1000 simulated chromosome II datasets. The red line represents the p = 0.01 significance threshold from the simulations.

We previously showed that the allele frequencies in the RIAILs depart from the neutral expectation, implicating selection during the application of the cross design [40].We extend that analysis here, estimating expected allele frequency skew using our simulations that explicitly incorporate marker spacing and recombination domain structure. Chromosome I (p<0.001) and chromosome II (p = 0.001) exhibit significant allele frequency departures from the neutral expectation (Figure 3). The other chromosomes exhibit allele frequencies consistent with neutrality (III, IV, V, X: p = 0.449, 0.213, 0.155, 0.323 for observing the largest allele frequency skew by chance).

In addition to selection on individual alleles, a more subtle form of selection is likely to operate in a cross of divergent selfing strains: epistatic selection to maintain coadapted combinations of alleles. Such selection should decrease the recombination fraction between coadapted loci without altering allele frequencies [70]. We compared the genetic lengths we observed for the RIAIL chromosomes to the expected genetic lengths determined by the RIAIL simulations, which employed 50 cM meioses and yielded expected lengths of approximately 300 cM for each autosome and 214 cM for the X chromosome. Chromosomes I, II, and III were shorter than expected in the absence of selection (one-sided p = 0.011, 0.002, 0.010, respectively; Figure 2B), while the others were not different from their expected lengths. For chromosomes I and II, the shortened genetic length is attributable at least in part to selection on single loci causing associated allele frequency skews. Chromosome III, however, is about 11% shorter than expected, despite no evidence of selection altering single-locus allele frequencies and no sign of distortion relative to the WormBase map of chromosome III (Figure S1).

The simulations were performed under the assumption that male meiosis is identical to hermaphrodite meiosis, and that oogenic and spermatogenic meioses within hermaphrodites are identical, with exactly one cross-over per chromosome per meiosis. That chromosomes IV and V exhibited the expected lengths suggests that the different settings for meiosis do not alter global crossover rates, although we cannot test sex-differences in local patterns of recombination frequencies.

We next sought evidence for epistatic selection generating associations between alleles on different chromosomes [70],[71]. We calculated p-values for Fisher's Exact Test for the 877,079 pairs of non-syntenic SNPs and found that the distribution of p-values is uniform; 1.2% of tests were significant at p<0.01, and 0.09% were significant at p<0.001. No tests were significant at the Bonferroni-corrected threshold. An analysis of the false discovery rate, based on permutations of genotypes by chromosome, found no threshold at which the FDR fell below 0.5. The maximum observed *r^2^* between nonsyntenic sites was 0.087, demonstrating the absence of strong correlations among chromosomes.

### Recombination Rate Modifiers

Segregating modifiers of recombination rate may influence the number or distribution of recombination breakpoints in the genomes of recombinant inbred lines [72],[73]. Such modifiers may be detected as QTLs for breakpoint number. We counted the breakpoints on each chromosome and mapped the number as a quantitative trait using structured nonparametric interval mapping [74]–[76]. The total number of breakpoints varies among the RIAILs from 6 to 29 with mean 15.

**Figure 4:**
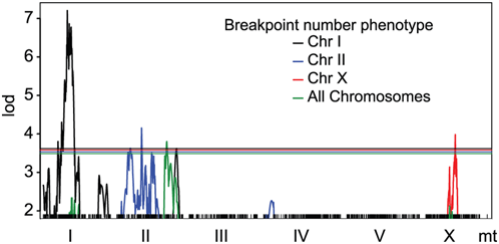
Breakpoint counts exhibit linkage to genomic intervals. Lod scores from nonparametric interval mapping are plotted as a function of genetic position for the four breakpoint count traits that exhibit significant linkage. Horizontal lines represent trait-specific genome-wide significance thresholds (p = 0.05) estimated by structured permutation.

Total breakpoint count links significantly to chromosome II (lod = 3.80, genome-wide p = 0.026; Figure 4). The Hawaii allele of the QTL is associated with slightly higher breakpoint numbers on every chromosome.

**Figure 5:**
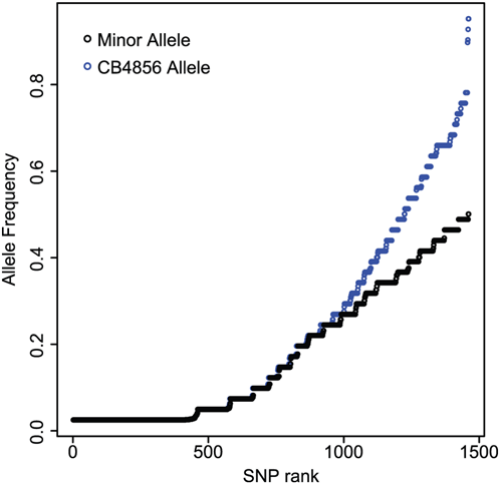
The Hawaiian isolate CB4856 has a large excess of rare alleles. Each of 1460 SNPs is plotted according to the frequency of the minor allele (black) or the frequency of the CB4856 allele (blue). Under panmixis, our SNP ascertainment should cause both sets of points to fall on straight lines, connecting allele frequencies 1/41 and 20/41 for minor allele frequency and 1/41 and 40/41 for Hawaii allele frequency. The plot shows that there is a large excess of rare alleles and that these rare alleles are CB4856 alleles.

Meiosis in *C. elegans* involves regulatory proteins that are unique to individual chromosomes or pairs of chromosomes, raising the possibility that segregating modifiers of recombination may have effects limited to individual chromosomes [77]. Similarly, modifiers may act in *cis* to alter recombination probabilities. To address these possibilities, we considered the number of breakpoints on each chromosome separately (Figure 5). Chromosome I breakpoint number exhibited a very significant linkage to chromosome I (lod 7.21, genome-wide p<0.001 by structured permutation). A second QTL, located on chromosome II, reached nominal genome-wide significance (lod 3.62, p = 0.050). Chromosome II breakpoint number exhibited significant linkage to chromosome II (lod = 4.154, p = 0.008), and X chromosome breakpoint number linked to the X chromosome (lod = 3.98, p = 0.022). Breakpoint number on chromosomes III, IV, and V did not link to any QTLs, even at the less stringent p-values required for significance at the chromosome-wide level.

The linkages of chromosome I and II breakpoint number to their own chromosomes are readily interpreted as artifacts attributable to unbalanced allele frequencies shaped by selection (Figure 3). As selection drives one allele to low frequency, the presence at a nearby locus of the allele from the same donor increases the probability that a breakpoint will be present between the two loci. Thus a selective sweep causes an association of linked alleles from the disfavored genome with high recombination breakpoint numbers. In our cross, presence of a Hawaii allele in the center of chromosome 1 all but guarantees the presence of a breakpoint to its left, due to the strong selection against Hawaii alleles at the gene *zeel-1*.

### Haplotype Diversity and Population History of Wild Isolates

Genotype data from 1460 N2-CB4856 SNPs (Table S2) distinguished only 41 haplotypes among 125 wild isolates (Table S3). Of the 1460 loci assayed, 101 exhibited genotyping failures in one or more wild isolates, consistent with deletions or SNPs in those strains interfering with the genotyping assay. These genotyping failures exhibit significant LD with adjacent SNPs, and in strain JU258, where large deletions have been identified by tiling array experiments [53], 15 of the 25 such calls fall within deletion predictions. The segregating genotyping failures are dramatically overrepresented on the chromosome tips and arms, particularly IIL and VR (Table 2), and depleted from the centers (Fisher's exact test, p = 10^−13^). Despite the additional information provided by these putative deletion genotypes, they distinguished only two haplotypes otherwise identical according to SNP genotypes.

**Table 2:** Distribution of Putative Deletions in Wild Isolates.

Chr	left tip	left arm	center	right arm	right tip
I	0/7	2/47	0/103	7/58	0/3
II	0/3	18/67	1/104	4/40	1/9
III	4/9	5/47	3/89	2/43	0/9
IV	0/2	4/45	5/131	3/65	4/11
V	3/10	9/77	7/156	14/58	2/7
X	0/5	1/87	0/93	2/58	0/11
All	7/36 (19%)	39/370 (11%)	16/676 (2%)	32/322 (10%)	7/50 (14%)

Markers Segregating Putative Deletions/Total Markers.

Among wild isolates from recent systematic collections, most haplotypes are confined to a single locality, though each locality may harbor multiple haplotypes (Table S3), as others have observed [23],[24],[45],[47],[50]. The only exceptions are haplotype 25, shared between Le Blanc and Hermanville in France [23] (∼310 km apart), and haplotype 40, shared between Mecklenbeck and Roxel in Germany [50] (∼5 km apart). Among the classical wild isolates from the CGC, a collection assembled without systematic sampling, SNP haplotypes are often shared among distant localities. Haplotype 1 is shared by N2, from Bristol, England, PX176 from Eugene, Oregon, and TR388 and TR389, from Madison, Wisconsin. Haplotype 19 is shared by AB2, from Adelaide, Australia, CB4855, from Palo Alto, California, and CB4858, from Pasadena, California. The similarities among classic strain haplotypes raise the possibility that these strains are not independent wild isolates, a point to which we return in the Discussion.

The SNPs are derived entirely from a comparison of N2 and CB4856 sequences, creating a strong ascertainment bias. In a panmictic population of constant size, ascertainment from a pair of chromosomes should bias the allele frequency spectrum observed in the rest of the population, yielding a uniform distribution [78]. In our data, the allele frequency is strongly skewed, with a dramatic excess of alleles observed only once (Figure 5). The skew is not consistent with a simple explanation in terms of population expansion, because the two alleles are not equally represented among the minor alleles. Instead, the allele found in CB4856 is almost always the rare allele (83% of sites; Figure 5). For 461 SNPs (32%), the Hawaii allele is unique to the Hawaiian strain, while no alleles are unique to Bristol, nor to haplotype 1. At two sites, only haplotypes 1 and 2 have the Bristol allele, and at just 12 of the 1460 sites is the Bristol allele found in fewer than 10 of the 41 haplotypes.

**Figure 6:**
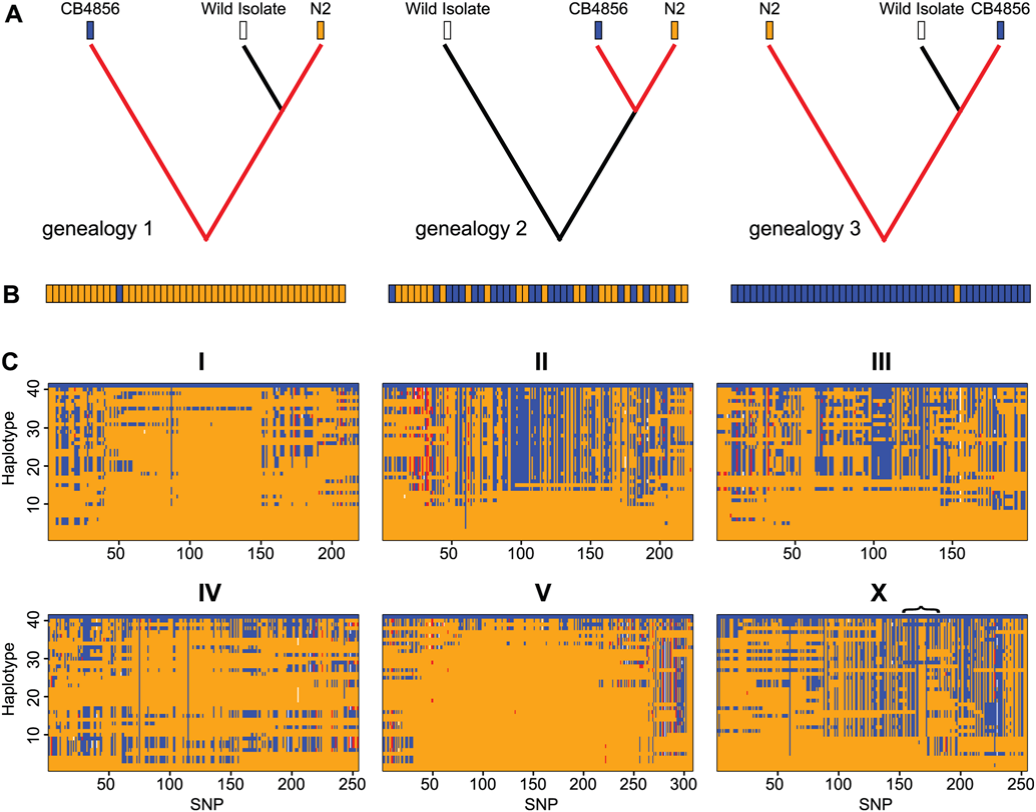
Wild isolate genomes. (A) The effects of SNP ascertainment on haplotypes. All SNPs were ascertained by comparing N2 and CB4856, and must therefore have arisen by mutation on the genealogical branches connecting those two strains. When a third strain is considered, there are three possible genealogies, but all SNP-generating mutations must reside on the ascertained branches, shown in red. The allelic states of the ascertained strains are shown as blue (CB4856) and orange (N2), and the wild isolate allele will be shared with either strain with probabilities that depend on the genealogy. (B) Expected wild isolate haplotypes from each of the genealogies under ascertainment. Typical haplotypes are represented as strings of SNP alleles colored by whether they are identical to N2 or to CB4856. In genealogies 1 and 3, most mutations will fall on the long outgroup branch, and the wild isolate will resemble the strain with which it shares a recent ancestor. In genealogy 2, the two ascertained branches have equal length with respect to the wild isolate, yielding an equal probability of each allele at each position. (C) Haplotypes of wild isolates. Each of the 41 distinguishable haplotypes is represented as a row for each chromosome. N2 carries haplotype 1 (all orange alleles) and CB4856 carries haplotype 41 (all blue). Putative deletions are red. The bracket above the X chromosome labels the interval across which haplotypes 29 and 39 exhibit haplotypes consistent with genealogy 3.

The excess of Bristol alleles is explained by the combination of ascertainment bias and population structure. The effects of these phenomema are revealed by the sequence of allelic states along the wild isolate chromosomes. Considering a single wild isolate and the two ascertainment strains, there are three possible genealogies for each nonrecombining segment of the genome (Figure 6A). Because we observe only those SNPs that arose as mutations on the branches connecting N2 and CB4856, the three genealogies predict distinct patterns of allelic states in the wild isolate genome (Figure 6B). Under panmixis, we should expect the genealogies to be equally common, but because our sample is conditioned on the presence of a SNP between N2 and CB4856, genealogies 1 and 3, which have more opportunity for such SNP-generating mutations to occur, should be overrepresented. In our data, however, the genealogy with CB4856 most closely related to the wild isolate (genealogy 3) appears to be absent (Figure 6C). Instead, the wild isolate chromosomes are mosaics of the other two genealogies, consistent with ongoing genetic exchange among such strains to the exclusion of the CB4856 lineage.

**Figure 7:**
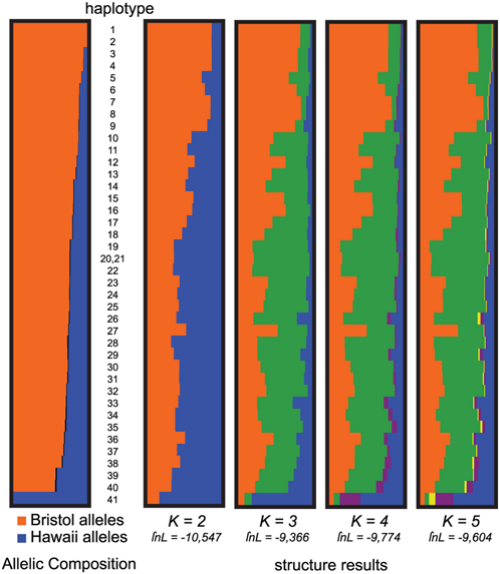
Population Structure. Distinguishable wild isolate haplotypes are represented as rows. At left, the allelic composition of each haplotype is represented by orange and blue bars. At right, population assignments from *structure* are shown for each haplotype, with the most N2-like ancestral population orange and the most CB4856-like blue. Likelihoods for alternative numbers of ancestral populations (*K*) are shown below each plot. At *K* = 1, lnL = −23076.

The excess of N2 alleles characterizes every strain (Figure 7); the least N2-like of the strains, haplotype 39 from the Portuguese island of Madeira and haplotype 40 from northern Germany, carry 58% and 57% N2 alleles (p<10^−7^ for each under the null hypothesis that alleles are equally likely, as expected in the absence of structure). The only evidence for recent genetic exchange involving CB4856 is the X chromosome of haplotypes 29 (MY1) and 39 (JU258), which share a run of 30 out of 31 CB4856 alleles (Figure 6C). Much of the rest of the MY1 X chromosome is highly N2-like, but the JU258 X chromosome contains a significant excess of CB4856 alleles (59%; p = 0.002), uniquely among all the wild isolate chromosomes.

The wild isolate chromosomes differ in their distributions of SNP genealogies (Figure 6C). The centers of chromosomes I and V, in particular, are almost entirely N2-like (genealogy 1) in the wild isolates, while the majority of wild isolate chromosomes exhibit outgroup-like (genealogy 2) haplotypes across the centers of chromosomes II and X. For almost every chromosome, at least one strain retains a chromosome whose haplotype is largely consistent with genealogy 2, in which N2 and CB4856 are more closely related to one another than to the wild strain (Table 3). These haplotypes represent repositories of allelic variation that exceeds that available in N2-CB4856 comparisons. For chromosome V, however, only one wild isolate has more than 40% CB4856 alleles, and most strains are entirely N2 through the center of the chromosome. The wild isolate carrying the least N2-like haplotype varies by chromosome, meaning that there is no single ‘next-best’ strain for SNP discovery genome-wide. The most useful strains for each chromosome are indicated in Table 3. Pairwise similarity among haplotypes is plotted in Figure S2.

**Table 3:** Divergent Chromosomal Haplotypes.

Chr	% N2 alleles in least N2-like haplotype	Least N2-like haplotype (locality)	Number of 41 haplotypes with <60% N2 alleles
I	50.2	35 (Germany)	1
II	44.6	19 (California)	22
III	47.2	26 (California)	14
IV	51.8	7,8 (France)	7
V	58.3	40 (Germany)	1
X	40.7	39 (Madeira)	6

We attempted to characterize the global population structure of *C. elegans* using the Bayesian approach of *structure* 2.2, which estimates the proportion of each strain's ancestry derived from each of a fixed number of ancestral populations [79],[80]. The analysis strongly favored multiple ancestral populations and conferred the highest likelihood on a population history involving three ancestral populations now extensively admixed (Figure 7). The ancestral populations correspond roughly to a Bristol-like strain, a Hawaii-like strain, and a third population. The proportions of ancestry inferred for each wild isolate correspond roughly to the fractions of each genotype drawing from the three genealogies possible given our SNP ascertainment scheme. Consequently, the CB4856 alleles present in the wild isolates largely represent recent shared ancestry not with CB4856 but with a common ancestor of both N2 and CB4856 (genealogy 2). To the extent that much genealogical information is missing in genomic regions characterized by genealogy 2, due to ascertainment bias, the interpretation of the third ancestral population inferred by *structure* is unclear.

### Recombination in the Wild

We calculated bounds on the minimum number of recombination events, *R_min_*, required to explain the haplotype data under the assumption that each mutation is unique (i.e. an infinite sites model)[81]. The lower bound on *R_min_* is 40 or greater for each chromosome and is 90 for chromosomes III and X (Table 4). These numbers are substantially higher than those calculated from previous data sets, reflecting the larger number of markers in our analysis.

**Table 4:** Estimates of *R_min_* Bounds and *ρ*/Mb (Standard Error).

Chr	*R_min_*	*ρ*/Mb		
	lower bound	Left arm	Center	Right Arm
	upper bound	(se)	(se)	(se)
I	53	4.69	0.24	1.43
	116	(0.41)	(0.07)	(0.18)
II	67	1.23	0.01	2.30
	143	(0.15)	(0.01)	(0.27)
III	90	4.00	0.40	3.91
	177	(0.39)	(0.03)	(0.39)
IV	63	2.73	0.29	0.21
	139	(0.24)	(0.02)	(0.05)
V	40	0.91	−0.04	1.66
	79	(0.11)	(0.01)	(0.19)
X	90	0.49	0.60	2.63
	177	(0.03)	(0.05)	(0.24)

To assess the global pattern of linkage disequilibrium, we calculated *r^2^* for each pair of sites on each chromosome, excluding sites with minor allele frequencies less than 0.1, and we made a rough estimate of *ρ*, the population effective recombination parameter, by nonlinear regression of *r^2^* on physical distance separating the sites. Sites exhibit high correlations across megabase scales and even among unlinked sites (Figure S3), consistent with findings from microsatellites [24],[50], AFLPs [23], SNPs [52],[82], and sequence data [45]–[47]. Considering all pairs of linked sites, *r^2^* decays to half its initial value over a distance of 3.3 Mb (Figure S4), an LD half-length orders of magnitude higher than observed in most obligately outcrossing species, including *Caenorhabditis remanei* [82], *Drosophila melanogaster* [83], and maize [84], which exhibit half-lengths measured in tens to hundreds of base pairs, and humans, where the number is in the tens of kb [85]. Even in *Arabidopsis thaliana* and rice, partial selfers like *C. elegans*, the LD half-length is measured in kb rather than Mb [86],[87].

**Figure 8:**
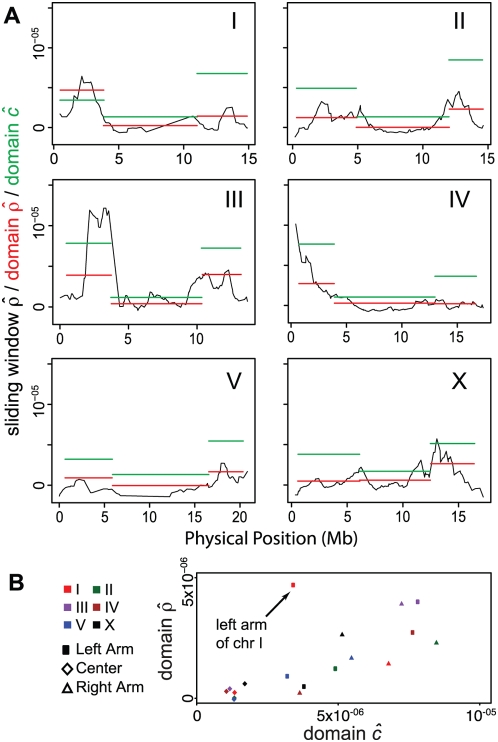
Population genetic and meiotic recombination rate estimates. (A) The population effective recombination parameter estimate 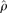 (per base pair) is plotted in black for sliding windows of 2 Mb centered on each SNP. Estimates are derived from the rate of decay of linkage disequilibrium with physical distance. Red bars indicate the estimates of 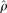 for whole recombination rate domains (arms and centers), and green bars indicate 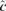, the estimated meiotic recombination rate per base pair, inferred for each domain from the recombination fraction observed in RIAILs (Figure 1; Table 1). (B) Domain-specific estimates of *ρ* and *c* are correlated, and 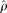 is about 40% the magnitude of 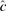.

To gain a finer-scale understanding of LD, we estimated *ρ* for 2 Mb windows centered on each SNP and for whole recombination rate domains (Figure 8A; Table 4). Variation in estimates of *ρ* along the chromosomes echoes the variation in recombination rates seen in the RIAILs, with 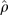 higher in arms and lower in centers. The similarity continues to the pattern of rate differences between the left and right arms of each chromosome, with the exception of chromosome I, where selection in the RIAILs resulted in a compressed genetic map on IL and where balancing selection at the same interval among wild strains may result in reduced LD and elevated estimates of *ρ* [40]. The half-lengths of LD for arm domains range from 500 kb (IL) to 9.9 Mb (IVR). The center-domain half-length is shortest on the X chromosome (3.5 Mb), while several chromosome centers exhibit no meaningful decay of LD with distance (IIC and VC). Treating each arm and center domain as an observation (Figure 8B), 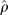 and recombination rate are well correlated (r = 0.692, p = 0.001; r = 0.860, p<10^−5^, when IL is excluded). The estimated population effective recombination rate is about 40% the meiotic recombination rate *c* estimated from the recombination fraction in the RIAILs, with the left arm of chromosome I a notable outlier.

**Figure 9:**
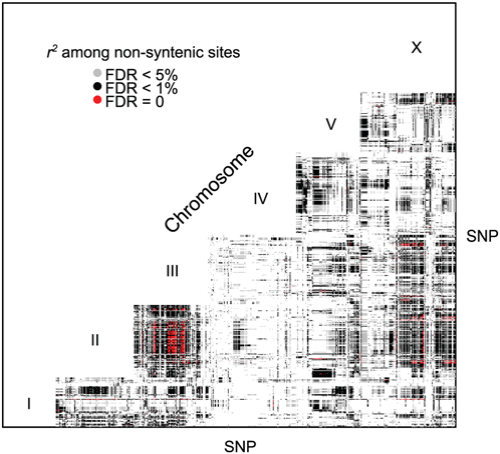
Linkage disequilibrium among unlinked sites. Every pair of unlinked SNPs with a significant *r^2^* at the specified false discovery rate is plotted. The axes represent the physically ordered SNPs spaced equally and not by distance.

Linkage disequilibrium extends among unlinked chromosomes. We calculated *r^2^* for all unlinked pairs of sites and found an excess of linkage disequilibrium across the entire range of *r^2^*. With a false discovery rate of 5%, 77,447 of 254,343 nonsyntenic pairs (30%) exhibited linkage disequilibrium, and 1918 pairs were in LD with zero false discoveries. Nonsyntenic associations extend primarily between chromosomes 2, 3, and X (Figure 9; Figure S2). In many strains these three chromosomes exhibit haplotypes consistent with genealogy 2 (Figure 6C), implying that both population structure and ascertainment bias may contribute to the elevated LD.

### Association Mapping

The potential to map loci at high resolution by association in wild *C. elegans* populations relies on appropriate levels of historical recombination to break correlation among markers while preserving correlations between markers and functional variants. To assess the utility of *C. elegans* for association mapping, we explored the correlations between the 907 non-singleton SNPs in out dataset that are not missing any data and two traits, copulatory plugging and epistatic embryonic lethality, that we have phenotyped in the wild isolates and whose underlying causative variants are known [40],[41].

**Figure 10:**
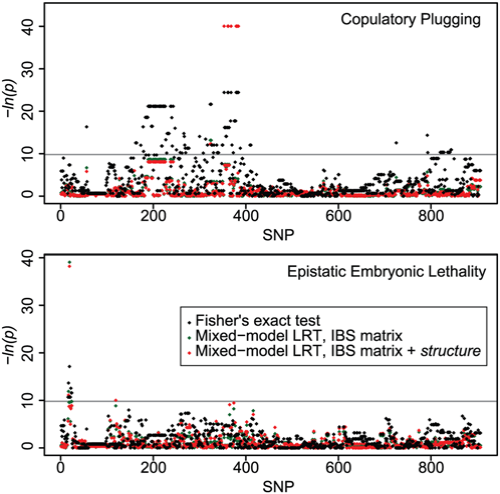
Association mapping in wild *C. elegans*. Negative log *p*-values for each of 907 SNPs are shown for two traits and three tests of association. SNPs are ordered by physical position from chromosome I through X. Gray lines represent Bonferonni-corrected *p* = 0.05 significance thresholds. In the upper plot, the highly significant red points cover identically positioned green points, and these points are plotted at an arbitrary −ln(*p*) of 40 because the perfect genotype-phenotype association yields an infinite −ln(*p*) under the mixed-model LRTs. The key applies to both panels.

By Fisher's exact test, 14% of all tested SNPs are significantly associated with copulatory plugging after Bonferroni correction for 907 tests (Figure 10). The known *plg-1* locus is on chromosome III [6],[41], where the most significant associations were observed, but significantly associated SNPs were also located on chromosomes I, II, and X.

Mixed-model approaches to control for family and population structure have been successful at identifying SNPs associated with traits in a background of high relatedness among strains [88],[89]. We incorporated pairwise similarity (identity-by-state, IBS) and admixture proportions estimated by *structure* into a mixed-model analysis using *EMMA* [88]. Only ten SNPs, all on chromosome III, remained associated with copulatory plugging in the mixed-model analysis incorporating the IBS matrix. Eight SNPs are in perfect LD with one another and with the trait; these SNPs are spread across roughly 2 Mb of chromosome spanning the causal locus at 8.86 Mb. Results were similar whether or not the *structure* results were incorporated into the analysis (Figure 10). The distribution of *p*-values from mixed-model analysis are nearly uniform (Figure S5), demonstrating the efficacy of the mixed-model approach for controlling background relatedness among strains.

The epistatic embryonic lethality involves two tightly linked genes mapping to the left side of chromosome I, with the two haplotypes maintained at intermediate frequency by balancing selection [40]. Fisher's exact test identified only 9 SNPs associated with the phenotype after Bonferroni correction, spanning 1 Mb centered on the causal insertion/deletion polymorphism at 2.35 Mb (Figure 10). The most highly associated SNP (p = 3.6×10^−8^), at 2,318,113, is 22 kb from the causal deletion. Mixed-model analysis incorporating only the pairwise identity matrix reduced the number of significant associations to just two, with the significance of the SNP at I∶2,318,113 dramatically increased. An additional SNP, very distantly linked at I∶12,967,075 is falsely weakly associated with the lethality phenotype when *structure* output is incorporated as an additional fixed effect in the model. Overall, however, the *p*-values at sites distant from the causal variant are nearly uniform (Figure S5).

## Discussion

### Recombination in *C. elegans* RIAILs

The genotype data from our 20-generation cross and from a global panel of wild isolates reveal the landscape of recombination and diversity across the *C. elegans* genome.

The RIAIL genotype data corroborate the domain structure of the *C. elegans* genetic map, with low recombination centers and high recombination arms, and we found the first clear evidence for recombination rate domains on the X chromosome. We used a segmented linear regression approach to estimate positions for the boundaries of the recombination rate domains. These boundaries show that the autosome centers are very similar to one another in relative size despite substantial differences in absolute size. The arms vary substantially in both absolute and relative size, and they vary substantially in recombination rate as well. Part of the variation among arms is explained by chromosome size, with shorter chromosomes forced to fit their obligatory crossover into a smaller physical distance.

All of the chromosomes exhibit large subtelomeric regions that effectively exclude nearly all recombination events. The tip domains, previously characterized as regions of high gene density on the basis of small genetic distances between mutations, are in fact physically large domains in which genes are almost perfectly linked. Overall we estimate that more than 7 Mb of the *C. elegans* genome (7%) falls in the tip domains of extremely low recombination. Despite the nonrecombining regions at the end of each chromosome, the RIAILs have a dramatically expanded genetic map and an expected mapping resolution of 225 kb, making them a useful tool for mapping QTL.

Two patterns confirm that local sequence features shape the recombinational landscape, despite the existence of potent mechanisms of chromosome-scale regulation of crossover events [90]. First, the low recombination central domains are not physically centered on the chromosomes, as would be expected if recombination rate is shaped merely by position in relative chromosomal coordinates. Second, the recombination rate variation we observe within domains, though not sufficient by itself to exclude constant-rate domains, is well mirrored by variation observed from the two- and three-point cross data compiled in WormBase. These repeatable patterns of small-scale rate variation establish that recombination is responsive to local variables.

Many questions about the *C. elegans* recombinational landscape remain unanswered. Each chromosome has one sharply defined arm-center boundary and one with a more gradual change in rate. The gradual boundary is closer to the pairing center on all but chromosome III, where neither boundary is as sharp as is typical for other chromosomesand where epistatic selection may distort the evidence of recombination rate variation. The role of temperature and sex in regulating crossover position also remains unclear, as our results, which include male and hermaphrodite meioses at 25°C, are similar to WormBase maps, derived primarily from hermaphrodite meioses at 20°C.

### Selection

Selection on the left arms of chromosomes I and II resulted in shorter than expected genetic maps, causing underestimation of meiotic recombination rates along those arms. Epistatic selection may also have compressed the genetic map of chromosome III. Epistatic selection may be common in *C. elegans*, because strains occur primarily as inbred, selfing lineages, within which coadapted alleles at unlinked loci have ample opportunity to arise and persist. Experimental data from laboratory crosses [40],[61] and from ecological genetics of natural populations [24] provide strong support for selection against recombinant chromosomes and interstrain hybrids.

Each mating during the RIAIL cross involved the random selection of an equal number of offspring, two, from each mating pair, giving the design the character of a selection-minimizing mutation accumulation experiment [91]. Consequently, selection must be extremely strong to have altered allele frequencies among the RIAILs. Moreover, because both N2 and CB4856 are viable strains with similar developmental rates, the selection must involve an interaction between alleles of the two strains. Strong epistatic selection clearly obtains in the chromosome I case, where paternal-effect-by-zygotic epistasis between tightly linked loci causes embryonic lethality [40]. Selection against the CB4856 alleles on IIL may be due to partially penetrant epistatic lethality or sterility, or possibly to a substantial growth rate defect such that the worms with the slow-growth genotypes remained early larvae at the time their more mature siblings were picked for subsequent crosses; growth rate variation is known to segregate in *C. briggsae* crosses [47]. The selected region of chromosome II, which spans the interval from roughly 0.5 Mb to 2.2 Mb, does not exhibit elevated linkage disequilibrium with other regions of the genome, which might be expected in the event of epistatic selection. One scenario is that the selected region, where CB4856 contains large deletions relative to N2 [53], may interact weakly with many regions of the genome, such that the interacting loci experienced little individual selection during the cross. The selected region is also strongly enriched for rapidly evolving F-box and MATH-domain genes, which exhibit evidence for positive selection in nature [92],[93], increasing the potential for coadaptation with other regions of the genome.

The shorter than expected map of chromosome III is not associated with allele frequency skew or apparent distortion of the recombination rate distribution compared to the WormBase map (Figure S1). There are four possible explanations for the observation. First, the short map may be due to chance (p = 0.010). Second, it may be due to epistatic selection involving multiple close pairs of sites, resulting in a short but proportionate map. Third, chromosome III may truly have a smaller genetic length than the other chromosomes. Both the WormBase map and data from other studies documenting the 50 cM length of chromosome III argue against this possibility [94]. Finally, the RIAIL map may truly be distorted but the WormBase map is erroneous in some details. The WormBase map derives from thousands of independent crosses performed over many decades in many labs, and the composite map may not accurately reflect the underlying recombination probabilities in any single cross.

We detected apparent QTLs accounting for recombination breakpoint number on chromosomes I and II that are clearly due to selection-driven allele frequency skew. Allele frequency skews are common in experimental crosses and attempts to map recombination modifiers must take them into account. Nevertheless, these skews lead only to false linkages of recombination modifiers to their own chromosomes (false *cis*-acting modifiers). We identified distant linkages for chromosome I breakpoint number and for total breakpoint number, and others have identified such distant linkages in other species [73]. These QTLs may represent true modifiers, but the strong evidence for highly constrained meiosis in *C. elegans*, with nearly complete interference [1], and the expectation that RIAIL designs will be poorly powered to detect modifiers [72] suggest that the approach of using breakpoint number to map recombination rate modifiers may suffer from additional unidentified biases.

### Population History of Wild Isolates

The 1460-SNP genotypes of 125 wild isolates represent only 41 distinct genome-wide haplotypes, consistent with the well-established prevalence of selfing among *C. elegans* in nature. Individuals from single localities are often genotypically identical, though we also observe substantial diversity among strains within localities.

The recent collections from France and Germany confirm that strains from different localities are typically distinct, with minor exceptions for proximate collections. Those results contrast with the pattern evident among the less systematically collected strains acquired over many years by the *Caenorhabditis* Genetics Center (CGC), where identical haplotypes are found among strains collected in far corners of the globe. The pattern suggests that these older collections may include strains whose origins are discordant with those implied by their locality data, perhaps as the result of sample mislabeling during their histories in the lab.

Recent findings by McGrath and colleagues [43] confirm these concerns. They determined that LSJ1, a strain maintained at a lab in California for decades, is most likely an early derivative of the same strain from Bristol that later gave rise to the laboratory strain N2, which carries haplotype 1. LSJ1 carries haplotype 2, which differs from N2 at just one SNP among the 1460 genotyped, but it also differs by functional mutations in two genes, *npr-1* and *glb-5* [43]. The N2 allele at these loci are present exclusively in strains of haplotypes 1–4, and the N2 *npr-1* allele occurs in all such strains with the exception of LSJ1. The implication is that the N2 mutations arose in the laboratory subsequent to the separation of the Bristol strain into its LSJ1 and N2 derivatives, and that strains carrying the *npr-1* and *glb-5* mutations are laboratory-derived descendents of N2.

Our genotype data corroborate documentary evidence suggesting that haplotypes 3 and 4 may be derived from laboratory crosses between N2 and a derivative of the Bergerac strain (haplotype 7), as foreseen by Egilmez et al. [48] on the basis of patterns of Tc1 transposon content. The likely laboratory origin of haplotypes 1–4 has several consequences. One is that all wild strains described from the Midwestern United States (TR388, TR389, and TR403) are dubious. Another is that the allelic variants cloned from haplotypes 1–4, including those in *npr-1*, *glb-5*, and perhaps *scd-2*, likely originated in the laboratory. Moreover, early inferences about *C. elegans* population biology may have been influenced by inclusion of multiple samples of similar laboratory strains as putative wild isolates from different geographic locations; of the 32 strains characterized for Tc1 patterns by Hodgkin and Doniach [6], 12 carry haplotypes 1–4.

Finally, the reliability of locality data from other early collections is called into question. A potential mixup involving the provenance of CB4555, DR1349, and CB4858, presumed derivatives of a strain from Pasadena, has been noted previously [6], and our data show CB4858 to be very distinct from CB4555 and DR1349, with the latter two carrying dubious haplotype 4 in common with strain DH424. We found that CB4858 shares haplotype 20 with strains from other localities, including AB2-4, from Adelaide, Australia, and CB4855, from Palo Alto, California. The genotypic similarity among CB4858, CB4855, and AB2-4, which has been noted previously [6],[45],[50], superficially suggests that they may share an ancestor in a laboratory. However, distinct chemoreceptor pseudogenization [59] and Tc1 patterns [6] provide evidence for the distinctness of CB4855 from the other strains, and AB4 and CB4858 appear quite distinct from one another in other SNP datasets [46],[52]. Our 1460 SNPs also fail to distinguish among recently collected strains known from other data to be distinct; for example our haplotype 33 includes strains known to vary at a microsatellite locus [50].

We used the RIAIL genotypes as a standard against which to evaluate wild isolate genotypes, and this control allowed us to identify 101 loci at which wild strains segregate alleles distinct from N2 and CB4856. Third alleles likely represent deletions overlapping the target SNP or imply the presence of additional SNPs that disrupt hybridization of the genotyping oligos. These variants are strongly enriched in chromosome arms and tips, particularly IIL and VR, previously identified as enriched in deletions based on hybridizations of genomic DNA to microarrays [53]. The elevated levels of putative deletion polymorphisms are not strictly attributable to recombination rate, as the levels are highest in the chromosome tips, which are very recombination poor. Variation among chromosomes also points to sequence-specific properties influencing these polymorphisms.

### Hawaiian Exceptionalism

*C. elegans* geneticists have long recognized that the Hawaiian strain, CB4856, collected from a pineapple field in 1972 [6], is divergent relative to other wild isolates [46],[52],[57],[59], with some loci dramatically diverged uniquely in this strain [46]. Our data confirm that CB4856 has experienced genetic isolation from all other sampled strains. The large excess of alleles unique to Hawaii, the excess of N2 alleles among all other strains, and the prevalence of two of the three possible genealogies for wild isolate chromosomes all point to the lack of recent reproductive contact between the population in which CB4856 resides and the remainder of the global *C. elegans* population. Every other wild isolate exhibits long stretches of N2-like alleles (genealogy 1; Figure 6), consistent with a recent common ancestor for N2 and the wild isolates for those regions of the genome. However, most wild isolates also carry large regions of genome consistent with genealogy 2, implying that these strains retain allelic variation beyond that present in the N2-CB4856 comparison. Consequently, the period of isolation of CB4856 must be short relative to the average coalescence time of *C. elegans* alleles. Population genetic analyses of resequencing data from selected genomic regions support the same conclusion; the Hawaiian strain is often nested well within the genealogy for particular loci [45]–[47]. The short period of isolation suggests that hyperdivergent sequences unique to the Hawaiian strain may represent targets of positive selection in Hawaii [46] rather than evidence for ancient divergence between lineages.

Stronger inferences about *C. elegans* population history are confounded by a severe and unusual SNP ascertainment problem, intermediate between phylogenetic ascertainment bias [95] and population genetic ascertainment bias [78]. The problem is worsened by the presence of population structure [96],[97], a variable whose effect on ascertainment bias depends on the nature of the structure, which is unknown in this case. The striking variation among chromosomes in haplotype patterns (Figure 6) may represent differences among chromosomes in the recency of common ancestry between N2 and CB4856, influencing the probability of observing genealogy 2 in wild isolates, or it may represent true differences among chromosomes in the prevalence of genealogy 2, due perhaps to selection. One reassuring observation is a strong qualitative correspondence between the haplotype pattern we observe for CB4858 and the genomewide SNP density between N2 and CB4858 inferred from whole genome resequencing [51]. The correspondence implies that our genealogical model of haplotypes from ascertained SNPs accurately reflects SNP density independent of N2-CB4856 divergence. The excess of genealogy 1 through the center of chromosome V among nearly all wild isolates may therefore represent a selective sweep favoring an N2 allele.

As all wild isolates should be similarly affected by ascertainment bias, we can infer that the relative divergence of JU258, a strain from Madeira, is not attributable to its origin from an island, as is sometimes supposed. Several strains from Northern Germany (e.g., MY2) exhibit similarly divergent haplotypes. At the same time, JU258 is unique among wild isolates in carrying a chromosome with a significant excess of CB4856 alleles, consistent with very modest reproductive contact between ancestors of those strains subsequent to the apparent isolation of CB4856 from all others [53],[59].

### Outcrossing and Recombination in Nature

Estimates of the frequency of outcrossing in wild *C. elegans* vary substantially [23],[24],[45],[55],[98], but all estimates derived from patterns of linkage disequilibrium point to very low rates. The first evidence for recombination among wild chromosomes appeared only in 2000 [52], and as recently as 2003 it was possible to invoke a single outcrossing event to explain *C. elegans* genotype data [46]. Our much denser dataset finds support for a large number of recombination events, with a minimum of 90 events required to explain variation on each of chromosome III and X.

Despite the evidence for ample recombination, linkage disequilibrium is high within and among *C. elegans* chromosomes. Our estimate of the population effective recombination parameter is strongly correlated with our estimate of recombination rate from the RIAILs, much more than is observed in *Arabidopsis thaliana* [86], although the scale over which rates are estimated may influence these analyses.

Strikingly, the magnitude of 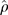 is only about 40% that of 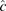, the estimated meiotic recombination rate. In a random sample of chromosomes, in the absence of ascertainment bias and population structure, 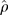 is an estimator of 4*N_e_c*(1-*s*) [99], where *N_e_* is the effective population size and *s* is the selfing rate. The effects of ascertainment bias and population structure prevent rigorous quantitative inference from our estimate of *ρ*; simple ascertainment bias is expected to elevate *r^2^* [100], but confounding structure irremediably complicates the matter. Supposing that our estimate reflects biological phenomena and not merely statistical artifact, there are two general explanations for the extremely low value of 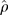. First, we may infer that the effective population size is very small and that the selfing rate is very large. Both *s* and *N_e_* have to be at the extremes of biological plausibility for this model to fit the observed relationship between 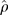 and 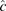, such that the product of the population size and outcrossing rate (1-*s*) is roughly 0.1. For example, the effective population size estimated from nucleotide polymorphism level 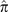, 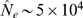 (at equilibrium, *π* = 4 *N_e_μ*; empirically, 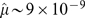 from mutation accumulation experiments[101] and 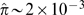 from population resequencing [45]), implies a low outcrossing rate of ∼2×10^−6^. Although this very rough estimate of outcrossing rate is less than an order of magnitude smaller than other estimates based on linkage disequilibrium in *C. elegans* [23],[45], direct estimates of outcrossing from heterozygote frequencies are much higher, in the range of 10^−2^ and greater [24],[55]. These direct estimates, in conjunction with our estimate of *ρ*, imply an effective population size smaller than 10. The disconnect between population genetic and direct estimates of outcrossing rates yields a second explanation for the low population effective recombination rate — selection against outcross progeny or recombinant genotypes, i.e., outbreeding depression [24],[61]. Heterozygotes produced by outcrossing may have low reproductive success and their offspring, with recombinant genotypes, may experience epistatic selection against deleterious combinations of alleles [24]. Outbreeding depression has been observed repeatedly in the laboratory [40],[61], including in the genotypic patterns evinced by the RIAILs on chromosomes I, II, and III. Moreover, a longitudinal study of wild populations of *C. elegans* provided strong evidence of selection against recombinant genotypes in nature [24]. That selection can influence 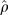 is evidenced by the elevated estimate on the left arm of chromosome I, where *zeel-1/peel-1* haplotypes are maintained by balancing selection [40].

Outbreeding depression may explain some of the strong linkage disequilibrium among unlinked sites (Figure 9), as epistatic selection against recombinants can preserve correlations among chromosomes. Because such patterns of LD among chromosomes are expected in the presence of population structure, however, strong inferences about the causes of LD are not possible.

### Association Mapping

Despite the exceptional levels of linkage disequilibrium across the *C. elegans* genome, we have demonstrated the feasibility of mapping common, large-effect variants by association. Ordinary correlations between alleles and phenotypes resulted in large numbers of false positive associations, but use of a mixed-model approach to control for background similarity among strains [88],[89] was successful.

The two traits we mapped, copulatory plugging and embryonic lethality, are best case scenarios for association mapping, with intermediate frequencies and Mendelian inheritance. Even in these cases, associations in regions of high LD necessarily span large intervals, more than 2 Mb in the case of *plg-1*. High-resolution association mapping in the *C. elegans* isolates collected to date is most likely to be fruitful for associations with markers on chromosome arms.

The very-high-resolution (∼20 kb) association detected for embryonic lethality reflects the exceptionally low LD around the loci responsible for the trait, attributable to the long-term maintenance of the alleles by balancing selection. The low LD around *zeel-1* and *peel-1* further confirms that the alleles are ancient and not involved in genome-wide differentiation between the two incompatibility classes [40].

### Conclusion

We have used high-density SNP genotyping to extensively characterize patterns of recombination in a large panel of C. *elegans* recombinant inbred advanced intercross lines. These lines provide a powerful permanent resource for high-resolution genetic mapping of phenotypic variation. We also genotyped a large collection of wild isolates, allowing us to define a set of isolates with distinct haplotypes and to describe in detail the genetic history of the C. *elegans* population. These results call into question commonly held beliefs about the origins of a number of isolates. Further insights into *C. elegans* population biology await broader surveys of sequence variation among the isolates.

## Methods

### Generating RIAILs

We generated recombinant inbred advanced intercross lines [102] from a cross between N2 and CB4856. We performed reciprocal crosses, yielding two classes each of male and hermaphrodite progeny differing in their mitochondrial and X chromosomes. We performed each of the four possible crosses among these strains, yielding four classes of F2 hermaphrodites and a single class of F2 males, ignoring the male mitochondrial genome, which is not transmitted. We performed the four possible crosses among these F2s, with each class of cross contributing 64 male and 64 hermaphrodite worms to the 512-worm F3 population, at which point we initiated random pair mating with equal contributions of each pair to each generation [62]. The random pair mating continued until the tenth generation.

Each cross plate contained a single male and a single hermaphrodite, and each generation some crosses failed due to poor male mating, evident from the absence of male offspring among the progeny. Other crosses failed due to segregating sterility, as evidenced by the failure of the hermaphrodite to produce any offspring. In addition, in some cases crosses failed because worms crawled to the edge of the plate and desiccated. To expand the population, we derived two lines from each plate containing tenth generation hermaphrodites. Each of the lines was then propagated by selfing a randomly selected hermaphrodite for each of 10 generations.

Worms were cultured using standard methods [103] and were maintained at 25°C during the construction of the RIAILs.

### Wild Isolate Strains

We acquired 125 wild isolates from three main sources.

Forty-three strains received from the *Caenorhabditis* Genetics Center come from unsystematic collections from sites in Europe, North America, and Australia since the 1940s. The origins of most of these strains are recounted in Hodgkin and Doniach [6], and the sources of the others (JU258, LSJ1, PB303, PB306, PX174, PX176, PX178, and PX179) are given in WormBase [67]. Two strains lack locality data. PB303 and PB306 were isolated by Scott Baird from isopods obtained from biological supply companies; the geographic origins of the isopods are unknown. LSJ1 derives from a laboratory in California, but it may represent an independent culture of the Bristol strain that gave rise to N2 [43]. The CGC received the strain in 1995.

The remaining wild isolates come from two systematic field collections. Haber et al. [50] collected 23 strains in northern Germany in 2002. We acquired these strains from the CGC. Barriere and Felix [23],[24] collected *C. elegans* from localities across France and we acquired from them 59 strains collected from 2001 through 2005.

### Genotyping

We collected DNA from each RIAIL and wild isolate using a salting-out protocol [104] applied to populations of each strain.

We genotyped the strains using Illumina's GoldenGate assay [105]. The assay interrogated 1536 loci reported in public databases as SNPs between N2 and CB4856. The databases contained 1099 confirmed SNPs and more than 17,000 SNPs predicted from sequence but not confirmed. 795 confirmed SNPs passed Illumina's design criteria. These were supplemented with 741 unconfirmed high-confidence SNPs with good design scores to make up the final set of 1536. This set was selected with the SNPdome algorithm (Illumina) to ensure uniform coverage of the *C. elegans* genome and to minimize gaps.

We used the RIAIL genotypes to validate the SNPs and confirm their map order. From the 1536 assay results, we identified 1205 high-quality SNPs with the following properties: N2 and CB4856 DNA samples were assigned different, homozygous genotypes with Illumina confidence scores >0.5; fewer than 5% of the 236 RIAILs had confidence scores <0.5; fewer than 2 RIAILs were called as heterozygotes. For these 1205 SNPs, we examined the wild isolates and assigned genotypes to calls with confidence scores >0.35. For the 285 SNPs that yielded some confidence scores between 0.35 and 0.5, fluorescence intensities were individually inspected and calls assigned manually when unambiguous.

For many of the 1205 RIAIL-confirmed SNPs, one or more wild isolates failed to give any genotyping signal. We identified a threshold of normalized intensities of both fluors ≤0.009 at which 768 wild isolate genotypes gave no signal (0.5018% of all calls) while the RIAILs gave only 8 genotypes at the same level (0.0028%), a 180-fold enrichment for the wild isolates. As these failed wild isolate genotypes exhibit linkage disequilibrium with well-genotyped SNPs, they likely represent mutations that disrupt the hybridization of the Illumina oligos to the genotyping interval. We assigned a third-allele call to these genotypes.

The remaining 331 SNP assays were individually examined to assign genotype calls. For 46 assays, N2 and CB4856 yielded the same genotype, implicating false-positive SNPs predictions. An additional 29 SNPs produced uninterpretable fluorescence intensity scatterplots. We were able to assign genotype calls for 196 SNPs which failed to pass the confidence threshold due primarily to low intensity. The remaining 70 SNPs exhibited more than two clusters of genotypes in plots of fluorescence intensities. We found that the extra clusters were due to hybridization of the SNP-assay oligos to additional loci which themselves exhibited segregation. As a result, each cluster could be assigned a homozygous genotype call on the basis of linkage disequilibrium with adjacent SNPs among the RIAILs.

The final dataset included 1460 SNPs. We excluded one RIAIL from subsequent analysis because its genotypes included a large proportion of ambiguous calls. The resulting dataset includes 236 RIAILs and 125 wild isolates scored at 1460 SNPs. The 527,061 genotypes include 1450 third allele (putative deletion) calls among the wild isolates, 654 Ns for bad data, and 180 heterozygote calls. Eight of the RIAILs exhibited short tracts of residual heterozygosity.

The mitochondrial genotype for each RIAIL was determined by PCR-RFLP, using primers 5′-ctcggcaatttatcgcttgt and 5′- cttactcccctttgggcaat and digesting with PmeI.

We estimated a genetic map for the RIAIL cross using *r/qtl* [74] and found that 6 SNPs had expected physical positions on chromosomes other than those to which they mapped. These may represent errors in the genome assembly or in oligo production; the oligo sequences map uniquely in the genome assembly. The expected and mapped physical positions of these SNPs are in Table S4. Analyses of RIAILs employed the 1454 physically mapped SNPs; the complete dataset is provided in Table S1. We considered the mismapped SNPs in analyses of WI haplotypes but excluded them from analyses that required physical positions. The complete wild isolate dataset is provided in Table S2.

In all cases where a RIAIL genotype contained an allele from one strain flanked by alleles from the other parental strain (i.e., a single-marker segment), we re-examined the plots of fluorescence intensities to confirm the genotype call; such a pattern is expected for a genotyping error and can strongly bias estimates of map lengths and breakpoint counts [71].

We estimate bin size as the distance from the end of a chromosome to the midpoint of the first breakpoint-containing interval or as the distance between the midpoints of successive breakpoint-containing intervals. This approach ignores bins created by multiple independent breakpoints within a single interval and uses interval midpoints rather than outside markers to avoid overlapping bins. Expected bin size is the per-base-pair sum of the squares of the bin lengths [106].

### Recombination Rate Domain Analysis

We estimated genetic distances in *r/qtl* using the Haldane map function, treating observed recombination fractions as though they had been observed in a backcross. The marker density is sufficiently high that the exact form of map function employed has little effect on estimated genetic distances.

We defined the tip domains of each chromosome to include all markers between the chromosome ends and the first recombination breakpoint observed in the RIAILs. The midpoint of this most distal recombinant interval was chosen as the tip-arm domain boundary. The non-tip markers were included in a segmented linear regression analysis, using the *segmented* package in *R* [107], to identify arm-center domain boundaries. To estimate confidence intervals for the domain boundaries, we used simulations of the RIAIL chromosomes. We simulated 1000 RIAIL populations for each chromosome, using the known pedigree. Each gamete received a meiotic chromosome with 0 or 1 breakpoints (i.e., complete interference [4]), the position of the breakpoints determined by the relative recombination fractions of the centers and arms estimated from the RIAILs. The tips were specified to be non-recombining and the two arms of each chromosome were assigned equal recombination probabilities per base pair; that is, intra-chromosomal differences in rate between arms were not modeled. Each chromosome was simulated as a sequence of markers with one marker for every kilobase of chromosome. We then sampled markers at spacing defined by the genotyped SNPs, yielding a dataset of RIAIL chromosomes simulated with discrete, constant-rate recombination domains. We estimated domain boundaries for the simulated chromosomes by segmented linear regression. The 95% confidence intervals vary in size depending on the size of the chromosome and the difference in recombination probability between adjacent domains. On average the intervals span 1.1 Mb.

The simulated RIAIL chromosomes were also used to estimate expected allele frequency skews and expected genetic lengths for each of the chromosomes. The RIAIL allele frequencies at each marker were estimated using the *sim.geno* function in *r/qtl* [74] to infer missing data.

WormBase [67] genetic maps are derived from data available on June 7, 2008, for 4542 genes with experimentally determined map positions and known physical positions. As our analyses of these data are qualitative, we made no effort to screen these data for quality, as evident from several obviously mismapped data points in Figure S1.

### Breakpoint Count QTL Analysis

We performed non-parametric interval mapping [76] in *r/qtl* [74]. The RIAILs differ in their relatedness as a result of the derivation of two selfing lines from each 10^th^ generation intercross hermaphrodite. The paired lines exhibit substantially higher similarity (mean percent bases shared ±standard deviation, 69.6±11.4%) than unpaired lines (52.8±9.5%), so that background similarity could inflate lod scores at markers unlinked to QTLs. Moreover, the significance of the lod scores would be overestimated by conventional permutation, because the RIAILs are not exchangeable; permuted datasets would break the associations between genetically and phenotypically similar RIAILs [75],[108]. Note that the mean similarity among unpaired lines is greater than the expected 50% because of the influence of selection on allele frequencies during RIAIL construction. For this reason we have not used simulated genotypes [108] to assess QTL significance. Instead we used a structured analysis and structured permutations. We split the dataset into two subsets with each RIAIL pair split between the two. We performed linkage scans separately for the two subsets and summed the lod scores. We permuted the two subsets separately 1000 times to derive genome-wide significance estimates for each phenotype.

### Structure Analysis

Estimation of population structure used a dataset of 40 haplotypes (haplotype 21, which differs from haplotype 20 only by a single putative deletion allele, was excluded, as the analysis treats these genotypes as missing data) and 1454 SNPs. We ran *structure* 2.2 [80] ten times at each of five values of K, the number of ancestral populations. We used the linkage model [79] with a burn-in period of 10,000 replicates followed by 50,000 replicates to collect estimated parameters and likelihoods. The outputs of the repeated runs at each K were aligned using *CLUMPP* 1.1.1 [109] and Figure 8 generated using *distruct* 1.1 [110].

### Linkage Disequilibrium

We computed lower bounds on *R_min_* for each chromosome using *HapBound* and upper bounds using *SHRUB* [81]. We used a dataset with 1318 SNPs, after excluding all sites with missing data or putative deletion alleles.

We used *Haploview* 4.0 [111] to calculate *r^2^* between all pairs of the 1042 sites with minor allele frequencies greater than 0.1 in the 40-haplotype dataset. We used these *r^2^* values to estimate *ρ* per basepair and its standard error by nonlinear regression using equation 3 of Weir and Hill [112], implemented with the R function *nls*. This simple method of moments estimator roughly approximates a likelihood estimator. Estimates of the half-length of LD represent the distance at which the expected value of *r^2^* from the nonlinear regression drops below half its initial value. To estimate *ρ* in sliding windows, we used the *r^2^* values among SNPs within 1 Mb to either side of each focal SNP. These 2 Mb windows are the smallest practicable windows given our marker density. We also estimated *ρ* for whole arms and centers, using the domain boundaries estimated from the RIAILs and shown in Table 1.

We estimated the distribution of *r^2^* among nonsyntenic sites in the absence of association from 100 permutations of chromosomes among the 40 wild isolate haplotypes, preserving allele frequencies and chromosomal haplotype frequencies but breaking correlations among chromosomes. The means of the ranked nonsyntenic *r^2^*values across permutations provides an estimate of the number of false discoveries at each quantile of the *r^2^* distribution. Permutations and calculations were performed in R, and *r^2^* was calculated using the LDmat function in the *popgen* library (http://www.stats.ox.ac.uk/˜marchini/software.html). The dataset included 784 sites with no missing data and minor allele frequencies greater than 0.1.

### Association Mapping

We excluded singleton SNPs and those with missing data and used the resulting 40×907 matrix to estimate an identity-by-state kinship matrix using *EMMA* [88]. We did not remove SNPs in perfect linkage disequilibrium with other SNPs because we sought to discern the genomic extent of intervals associated with traits. We estimated the significance of associations in the mixed-model analysis using likelihood ratio tests with the function *emma.ML.LRT*, incorporating the kinship matrix and in some cases the ancestral population admixture assignments from *structure* (*K* = 3) as fixed effects.

## Supporting Information

Figure S1 RIAIL maps recapitulate classical marker mapping results. Chromosomal and regional rate variation patterns observed in the recombinant inbred advanced intercross lines (black points) are similar to those observed from thousands of two- and three-point mapping experiments reported in WormBase (red points). The RIAIL map distances represented here are scaled to yield 50 cM total lengths for each chromosome. The classical mapping data corroborate the great difference in rate between the left and right arms of chromosome IV, with an exceptionally high rate on IVL between roughly 1.0 and 2.4 Mb. At a sub-arm scale, we see corroboration for variation along IIL very clearly and IR, VR, and XL less so. Other regions that show variation in the WormBase map are not evident in the RIAIL map, notably IVR, VL, and XR. Nevertheless, our results support the claim of Barnes et al. [65] that the arms are not truly constant-rate regions.
(3.99 MB EPS)

Figure S2 Pairwise identity among wild isolate haplotypes. For each chromosome, pairwise allele-sharing between each haplotype is plotted below the diagonal. Above the diagonal we present results of the same analysis excluding all singleton SNPs, all of which are unique to CB4856 (haplotype 41).
(1.65 MB EPS)

Figure S3 Linkage disequilibrium within chromosomes. Pairwise *r* ^2^ values for all sites with minor allele frequencies >0.1 are plotted. The axes represent physical position along each chromosome. Pairs of sites with *r* ^2^>0.5 are in black and those with *r* ^2^>0.9 are red.
(0.06 MB PDF)

Figure S4 Decay of linkage disequilibrium. Each point plots *r* ^2^ for a pair of sites with minor allele frequencies >0.1, colored by chromosome, as a function of the physical distance between the two sites. The curves plot the nonlinear regression of *r* ^2^ on distance using the sample-size-corrected relationship between the variables from Weir and Hill [112].
(0.19 MB PDF)

Figure S5 Distributions of *p*-values for tests of association. The calculated *p*-value for each SNP marker is plotted under three tests of association as in Figure 10: Fisher's exact test, mixed-model likelihood ratio tests incorporating a genotypic similarity (IBS) matrix, and mixed-model LRT incorporating both genotypic similarity and the results of *structure* analysis. The straight line represents the expectation for uniformly distributed *p*-values. Without mixed-model control for genomic similarity, the *p*-value distribution is profoundly skewed to low values.
(4.04 MB PDF)

Table S1 SNPs and RIAIL Genotypes. SNP details and genotype data for 236 recombinant inbred advanced intercross lines.
(0.94 MB TXT)

Table S2 SNPs and Wild Isolate Genotypes. SNP details and genotype data for 125 wild isolates.
(0.62 MB TXT)

Table S3 Strains and their Haplotypes. Strain, haplotype number, locality, and counts of genotype calls.
(0.03 MB XLS)

Table S4 Misplaced SNP markers. Illumina oligo sequences, expected positions, and map-based positions.
(0.02 MB XLS)
