# Supplementary figures and images for: Recombinational Landscape and Population Genomics of Caenorhabditis elegans
Source: PLoS Genet. 2009 Mar 13;5(3):e1000419. doi: 10.1371/journal.pgen.1000419 (PMC2652117; doi:10.1371/journal.pgen.1000419)

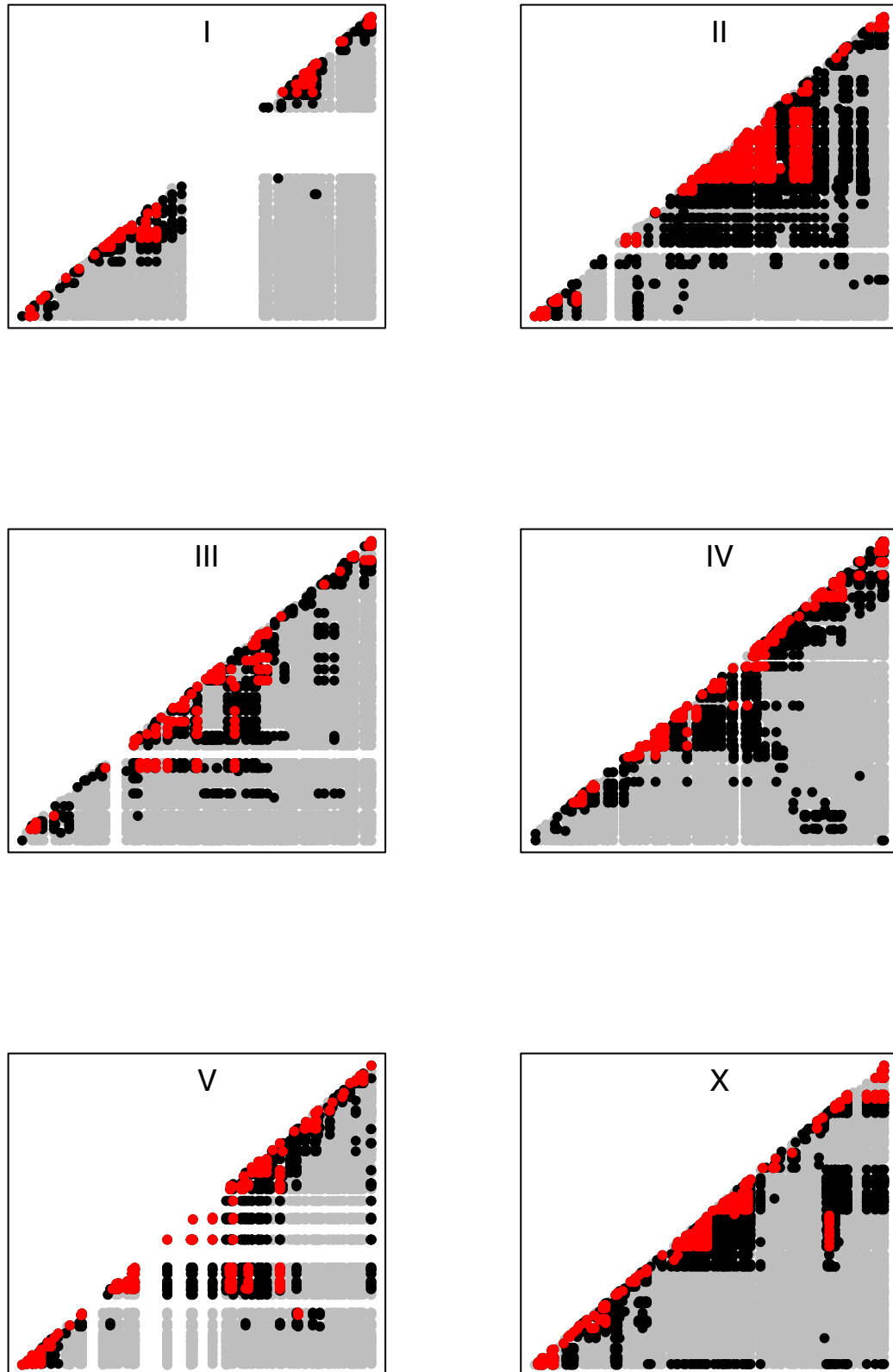

Figure S3

Supplement: Figure S3 — Linkage disequilibrium within chromosomes. Pairwise r 2 values for all sites with minor allele frequencies >0.1 are plotted. The axes represent physical position along each chromosome. Pairs of sites with r 2>0.5 are in black and those with r 2>0.9 are red. (0.06 MB PDF) [file pgen.1000419.s003.pdf]

Figure S4

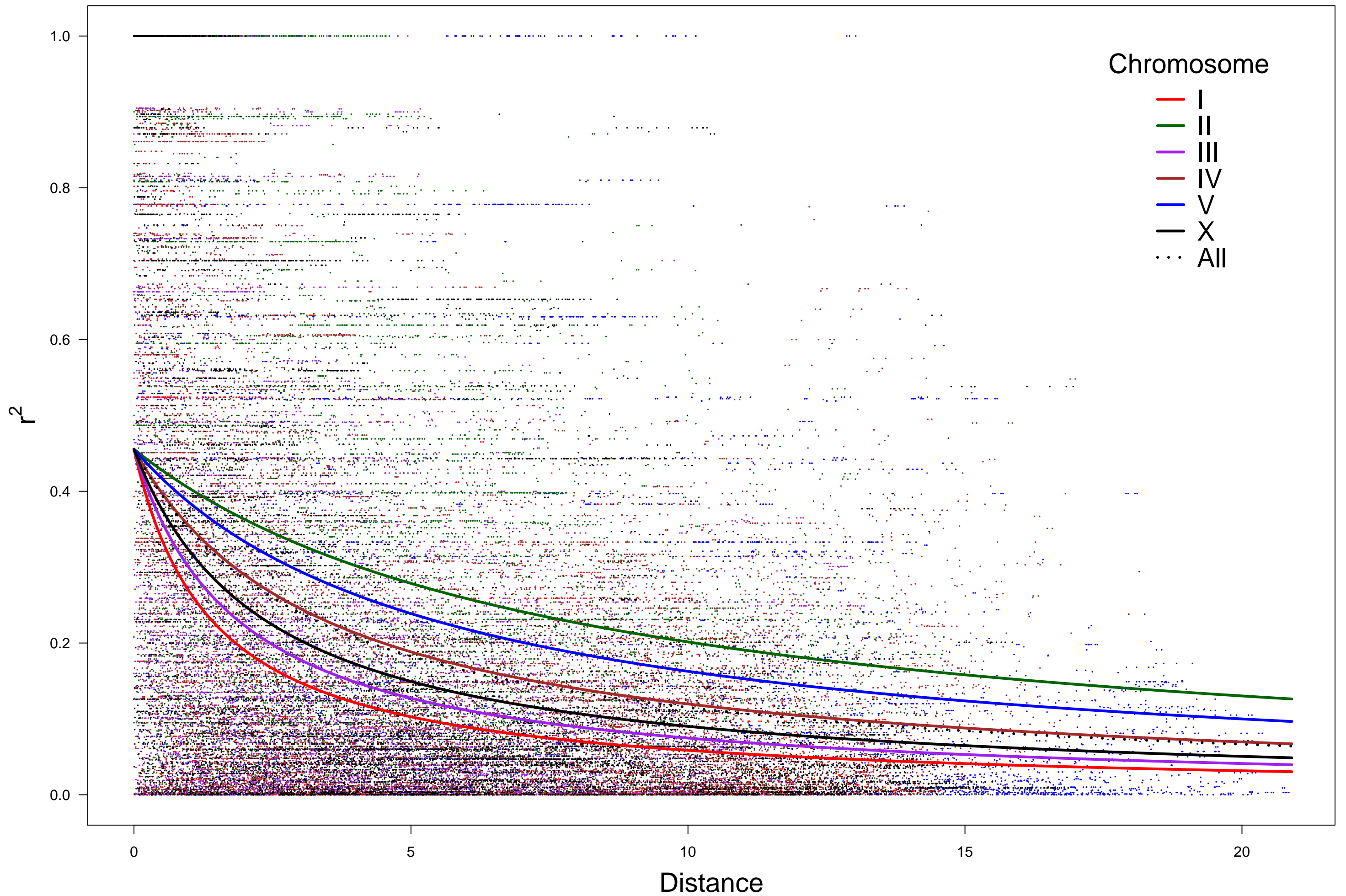

Supplement: Figure S4 — Decay of linkage disequilibrium. Each point plots r 2 for a pair of sites with minor allele frequencies >0.1, colored by chromosome, as a function of the physical distance between the two sites. The curves plot the nonlinear regression of r 2 on distance using the sample-size-corrected relationship between the variables from Weir and Hill [112]. (0.19 MB PDF) [file pgen.1000419.s004.pdf]

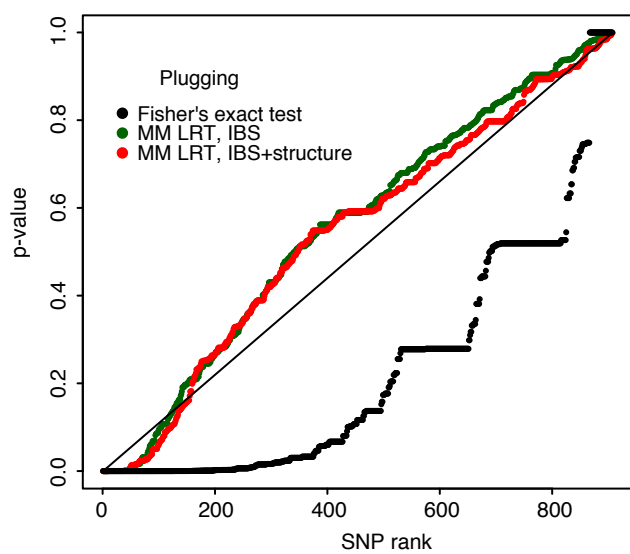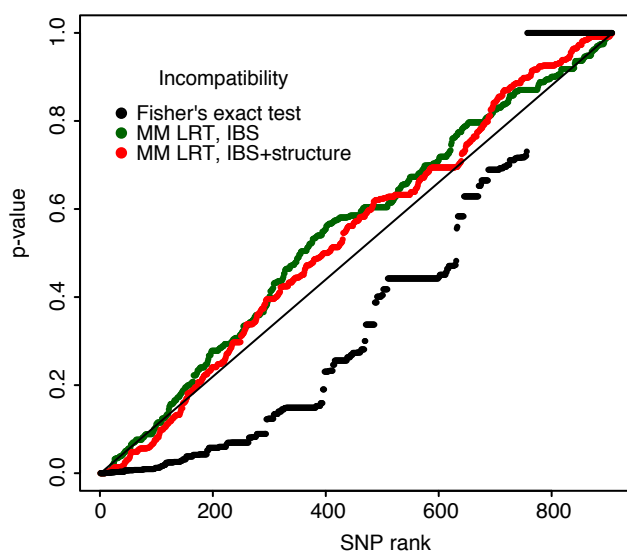

Figure S5

Supplement: Figure S5 — Distributions of p-values for tests of association. The calculated p-value for each SNP marker is plotted under three tests of association as in Figure 10: Fisher's exact test, mixed-model likelihood ratio tests incorporating a genotypic similarity (IBS) matrix, and mixed-model LRT incorporating both genotypic similarity and the results of structure analysis. The straight line represents the expectation for uniformly distributed p-values. Without mixed-model control for genomic similarity, the p-value distribution is profoundly skewed to low values. (4.04 MB PDF) [file pgen.1000419.s005.pdf]
